# Extracellular Nucleic Acids in the Diagnosis and Progression of Colorectal Cancer

**DOI:** 10.3390/cancers14153712

**Published:** 2022-07-29

**Authors:** Jakub Styk, Gergely Buglyó, Ondrej Pös, Ádám Csók, Beáta Soltész, Peter Lukasz, Vanda Repiská, Bálint Nagy, Tomáš Szemes

**Affiliations:** 1 Institute of Medical Biology, Genetics and Clinical Genetics, Faculty of Medicine, Comenius University, 811 08 Bratislava, Slovakia; vanda.repiska@fmed.uniba.sk; 2 Comenius University Science Park, Comenius University, 841 04 Bratislava, Slovakia; ondrejpos.sk@gmail.com (O.P.); nagy.balint@med.unideb.hu (B.N.); tomasszemes@gmail.com (T.S.); 3 Geneton Ltd., 841 04 Bratislava, Slovakia; 4 Department of Human Genetics, Faculty of Medicine, University of Debrecen, 4032 Debrecen, Hungary; gbuglyo80@gmail.com (G.B.); csok.adam@med.unideb.hu (Á.C.); soltesz.beata@med.unideb.hu (B.S.); 5 Department of Surgery, Transplantation and Gastroenterology, Semmelweis University, 1082 Budapest, Hungary; peter.lukasz@gmail.com; 6 Medirex Group Academy, n.p.o., 949 05 Nitra, Slovakia; 7 Department of Molecular Biology, Faculty of Natural Sciences, Comenius University, 842 05 Bratislava, Slovakia

**Keywords:** colorectal cancer, liquid biopsy, cell-free nucleic acids, biomarkers, non-invasive diagnosis

## Abstract

**Simple Summary:**

Colorectal cancer (CRC) is a disease that usually shows no evident clinical symptoms in the early stages, often leading to late diagnosis. Over the past few years, a new approach based on liquid biopsy has gained far-reaching applications in less-invasive CRC diagnosis and management, allowing for the use of extracellular nucleic acids as promising biomarkers to detect CRC at an early stage and monitor disease recurrence. That is why an up-to-date review and discussion of in-depth liquid biopsy-derived DNA and RNA biomarkers is essential. We hereby offer an overview of known predisposing genetic factors for developing sporadic and hereditary CRC, and an extensive repertoire of available extracellular DNA/RNA molecules with their potential clinical applications and shortcomings. Our review may be of value to experts dealing with CRC at the molecular level as well as to clinical professionals aiming for a better understanding of state-of-the-art techniques in CRC diagnosis and management.

**Abstract:**

Colorectal cancer (CRC) is the 3rd most common malignant neoplasm worldwide, with more than two million new cases diagnosed yearly. Despite increasing efforts in screening, many cases are still diagnosed at a late stage, when mortality is high. This paper briefly reviews known genetic causes of CRC (distinguishing between sporadic and familial forms) and discusses potential and confirmed nucleic acid biomarkers obtainable from liquid biopsies, classified by their molecular features, focusing on clinical relevance. We comment on advantageous aspects such as better patient compliance due to blood sampling being minimally invasive, the possibility to monitor mutation characteristics of sporadic and hereditary CRC in a disease showing genetic heterogeneity, and using up- or down-regulated circulating RNA markers to reveal metastasis or disease recurrence. Current difficulties and thoughts on some possible future directions are also discussed. We explore current evidence in the field pointing towards the introduction of personalized CRC management.

## 1. Introduction

Colorectal cancer (CRC) is one of the most threatening types of cancer in developed countries [1]. It is the 3rd most common malignant disease overall and the 2nd leading cause of death, with 2.3 million new cases per year registered worldwide [2]. Aging population, unfavorable eating habits, and other cumulative risk factors contribute to the growth of case numbers, while about 3–10% of cases are associated with inherited cancer predisposition (see Section 2). Based on tissue characteristics, five subtypes are known: adenocarcinoma (the most common subtype), carcinoid tumor, lymphoma, sarcoma [3] and gastrointestinal stromal tumor [4].

Screening families at-risk and the general population is essential, as early detection greatly improves prognosis. Available data clearly show the importance of early diagnosis, supported by the overall survival (OS) rate of CRC patients depending on the stage at which the primary tumor is diagnosed. Based on data from a retrospective population study [5], 5-year OS is 78.85% for the localized stage (stages I–II), 63.25% for the regional stage (stage III), and 20.31% for the metastatic stage (stage IV). Another study evaluating stage-dependent OS in CRC found 94%, 82%, 67%, and 11% for stages I, II, III, and IV, respectively [6,7]. 

Numerous traditional screening methods are available for CRC, including fecal occult blood test (FOBT) and colonoscopy. The latter is considered gold-standard, especially for inherited CRC syndromes such as Lynch syndrome (LS). Still, patient compliance is often poor, and a lack of quality standards results in missed cases [8]. FOBT is suggested for screening the general population and at-risk patients who reject colonoscopy, but its limitations include high false-positive rates and patient compliance dropping below 50% after 5–10 years [9,10]. Only about 40% of the population for whom screening is suggested will proceed with CRC testing [11]. Compliance would surely rise if more patient-friendly and less-invasive strategies were available. 

Methods introduced more recently include stool DNA testing (a highly sensitive method unaffected by the proximal location of the tumor but involving costs and technical difficulties associated with large-volume stool collection and transport, making it an unlikely candidate for a frequently performed mainstream screening method in the general population [12]) and ELISA-based screening tests for CRC antigens from blood samples. Testing of peripheral blood is a promising new direction, having the advantage of being much less invasive than colonoscopy and more convenient than stool testing, resulting in a much higher rate of patient compliance [13]. Nucleic acids are also readily detectable from blood; however, input costs must continue to be reduced, a process critical to the competitiveness of such strategies compared to conventionally used protein analyses [14]. Additionally, some mutations relevant for CRC therapy, such as the ones reported in the human epidermal growth factor receptor 2 (*HER2*) gene, are too small to be detectable at the protein level [15].

Some of the traditional tests are not invasive but are perceived as unpleasant by patients, suggesting the need for novel approaches to CRC screening. Liquid biopsy is acceptable for most patients and seems to be the best candidate in this context. In this review, we offer a brief overview of CRC genetics and discuss current liquid biopsy-derived DNA and RNA biomarkers showing promise in the screening of CRC in the general population and families at risk.

## 2. Genetics of CRC

Based on the etiology and the genetics of the disease, CRC is generally categorized into three groups: (i) sporadic, (ii) familial, and (iii) hereditary [16,17]. Sporadic cases represent about 75% of all incidences. Genetic factors still play a role, but only somatic mutations are present, and family members of affected individuals do not have an increased risk of developing the disease [18]. Familial CRC is not classified as hereditary and is frequently regarded as sporadic, as no causative genes have been identified yet. First-degree relatives of known patients are at higher risk compared to the general population [18,19]. Hereditary CRC is caused by a known mutation in the germ-line; the most common example is LS, but many other (rare) syndromic forms of CRC have been reported [17,20]. They are outlined in Table 1.

### 2.1. Hereditary CRC

LS is the most common hereditary colon cancer syndrome, also manifesting in other cancer types. It is responsible for 2–4% of CRC cases [37] and shows an autosomal dominant pattern of inheritance, caused by one of the DNA mismatch repair (MMR) genes being affected by a heterozygous germline mutation, or a deletion in the *EPCAM* gene [38], and the corresponding protein losing its function (and becoming undetectable by immunohistochemistry) following a somatic loss of heterozygosity in the colon tissue. The genes MutS homolog 2 (*MSH2*), MutL Homolog 1 (*MLH1*) and PMS1 Homolog 2 (*PMS2*) are involved in this type of repair mechanism [39], with *MSH2* and *MLH1* mutations having the greatest contribution to the development of LS-associated malignancies [40]. Tumors associated with LS show a high level of microsatellite instability (MSI-H), a feature not seen in the majority of sporadic CRC cases (see below) [41].

Other hereditary CRC syndromes worth noting include Peutz–Jeghers syndrome (PJS) caused by a mutation in Serine/threonine kinase 11 (*STK11*), usually presenting as multiple benign hamartomatous polyps [42,43], and MUTYH-associated polyposis (MAP) inherited in an autosomal recessive pattern characterized by biallelic germline mutations in MutY DNA glycosylase (*MUTYH*), a gene having a role in base excision repair (BER) [26,44]. Familial adenomatous polyposis (FAP), a well-studied disease with an autosomal dominant inheritance, has been reported to be caused by insertions and deletions in the adenomatous polyposis coli (*APC*) tumor suppressor gene [45]. Germline mutations of oncogene-induced senescence pathway genes cause serrated polyposis syndrome [46,47], usually presenting as multiple serrated polyps of the colon [46].

### 2.2. Sporadic CRC

Twelve percent of sporadic tumors are MSI-H, while the rest are classified as microsatellite stable (MSS) or show low level of microsatellite instability (MSI-L). Most sporadic MSI-H cases are associated with somatic MMR deficiency caused by the CpG Island Methylator phenotype (CIMP), in which the *MLH1* promoter shows biallelic somatic methylation with B-Raf Proto-Oncogene, Serine/Threonine Kinase (*BRAF*) mutations usually present in the background (almost never seen in LS) [41]. MSI-H tumors are thought to occur more proximally and are less differentiated, and usually show a better prognosis due to being sensitive to anti-programmed death receptor 1 (PD-1) therapy [48].

MSS tumors typically display chromosomal instability (CIN): numerical and structural alterations in chromosomes apart from a large variety of mutations in oncogenes and tumor suppressor genes [17]. Progressive telomere shortening occurs in the intestinal epithelium during aging in humans, and telomere dysfunction (anaphase bridging) has been documented in the adenoma-carcinoma transition, indicating that a telomere-based crisis may play a role in driving CIN in the early stages of human CRC [49]. Cancer progression is associated with telomerase reactivation, which is present in 85–90% of all cancer types, including CRC [50].

Mutations in *APC*, KRAS proto-oncogene GTPase (*KRAS*), and Tumor Protein 53 (*TP53*) are associated with lymph node metastasis [51]. *KRAS* mutations are found in about half of MSS/MSI-L tumors and make them resistant against anti-epidermal growth factor receptor (*EGFR*) antibody therapy. However, a fraction of wild-type *KRAS* tumors also display a poor response. The simultaneous presence of *KRAS* and *BRAF* mutations is rarely detected (most such cases are MSS CRC) [52]. 

## 3. Cell-Free Nucleic Acids as CRC Biomarkers

Liquid biopsy-based methods of cancer detection have undergone a substantial increase in popularity in recent years. Painless and quick sampling may help involve more average-risk people in cancer screening programs. As the carcinogenesis of CRC takes years, there is a relatively wide window available for early detection, but blood biopsies are likely to prove equally useful in the follow-up of non-metastatic and metastatic CRC (mCRC) [53,54]. On the other hand, circulating tumor DNA (ctDNA) assays do not provide insight into cell differentiation (tumor grade) or cancer stage (e.g., TNM classification), but suggest the overall tumor burden and may reveal the tumor’s molecular subtype and heterogeneity. So, liquid biopsy may not replace a tissue-based diagnosis, but rather provides alternate sampling for molecular tests.

**Figure 1:**
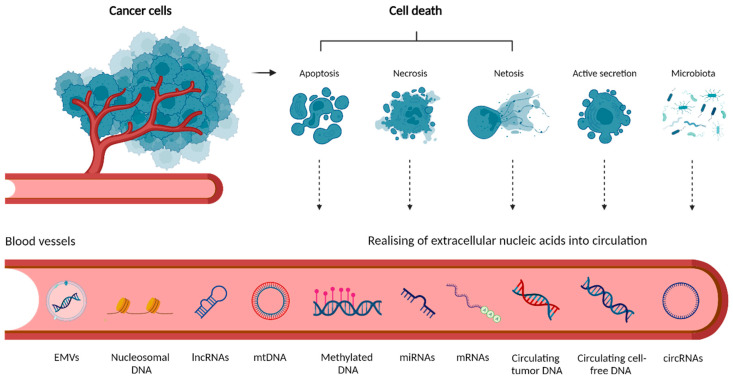
**Origin of extracellular nucleic acids in circulation.** Tumor cells release extracellular cfNAs through a combination of (i) cell death such as apoptosis, necrosis, and netosis or (ii) active secretion. Circulating bacterial DNA (cbDNA) may also be detected in blood samples from cancer patients. The loss of membrane integrity in necrosis results in releasing intracellular contents, including fragments of DNA, into the circulation. In netosis, activated neutrophils release neutrophil extracellular traps, webs of chromatin, and mitochondrial DNA (mtDNA). cfDNA may exist in the peripheral blood as either free or linked to proteins in the form of nucleosomal DNA, or associated with extracellular membrane vesicles (EMVs) such as exosomes or microvesicles secreted by cells. Methylated DNA and various types of RNA are present as well (created with Biorender.com, accessed on 21 July 2022).

There are five main sources of cell-free nucleic acids (cfNAs) in the circulation described until now: (i) apoptosis, (ii) necrosis, (iii) netosis, (iv) active secretion and (v) release by microbiota (Figure 1). cfNAs may freely circulate in body fluids, or may be bound to protein complexes or encapsulated in extracellular membrane vesicles (EMVs) (e.g., apoptotic bodies, microvesicles, or exosomes). EMVs mediate intercellular communication and were shown to play essential roles in the pathogenesis of CRC, as they are implicated in tumorigenesis, CRC progression, chemotherapy resistance, and metastasis [55,56]. Details of individual cfNA types and their release mechanisms were comprehensively covered in previous works [57,58,59,60,61] and are not the focus [55,56,57,58,59] of this review.

### 3.1. cfDNA Biomarkers

In response to the current status quo in the continuum of CRC management, the Colon and Rectal–Anal Task Forces of the United States National Cancer Institute recently convened a panel of multidisciplinary experts to summarize current data on the utility of ctDNA. They provide guidance and promote the efficient development and integration of this technology into clinical care. The panel focused on key areas in which ctDNA has the potential to change clinical practice [62].

The concentration of tumor-derived cfDNA, also known as ctDNA in the plasma of rectal and colon cancer patients, was compared in multiple studies, with varying results. Frattini et al. reported colon cancer patients having higher ctDNA concentrations than patients with rectal cancer (colon: 500 ng/mL, rectal: 250 ng/mL in plasma) [63], while Cassinotti et al. observed the opposite [64]. However, there is consensus on the usefulness of ctDNA biomarkers in CRC, as they show promise in the initial diagnosis, monitoring minimal residual disease, evaluation of treatment response in metastasis, identifying drivers of treatment sensitivity and resistance, and guiding therapeutic strategies to overcome resistance (Table 2) [53,65].

It has been demonstrated that cfDNA fragmentation profiles are different between healthy individuals and cancer patients [73] and also vary between tumor types [68]. Thus, accumulating evidence suggests fragmentomic cfDNA features as a potential cancer biomarker (Table 2). Zhitnyuk et al. were able to reveal the presence of early-stage colorectal and renal cancer with an area under the ROC curve (AUC) of 0.94 by deep targeted profiling of cfDNA end distributions and sequence motifs [66]. Moreover, since fragmentation is related to nucleosomal patterns, it may be useful for determining the source of tumor-derived cfDNA [67]. However, cfDNA fragmentation may not always be suitable to distinguish cancer from other forms of tissue damage. In this context, trauma-induced cfDNA was studied, while the concentration of short but not long cfDNA fragments was shown to be increased postoperatively in colorectal and bladder cancer [74].

CRC-specific mutations, including the ones in *APC, BRAF*, *KRAS* and *TP53*, seem to be equally detectable from cancer tissue and blood plasma [69]. Monitoring the presence of mutations observed at the time of diagnosis from ctDNA during the postoperative period may be used to predict CRC recurrence irrespective of the type of chemotherapy being applied (Table 2) [75].

Vidal et al. have demonstrated an assay offering a minimally invasive and highly sensitive method for RAS assessment in the plasma of mCRC patients, which may be readily implemented into routine clinical practice to perform baseline diagnosis to select candidate patients for anti-EGFR therapy. Moreover, a potential use in assessing the dynamics of *RAS* to monitor response and resistance to treatment has been suggested (Table 2) [71]. Another example is *HER2*, a well-known oncogenic driver in different tumor types [76]. Although its alterations are not common in CRC (3–5% of mCRC cases), when present, anti-HER2 therapy is an option [15]. Nakamura et al. reported that baseline ctDNA genotyping of *HER2* copy number may stratify patients according to the efficiency of therapy with an accuracy comparable to tissue genotyping. Since ctDNA genotyping can identify patients who benefit from dual-HER2 blockade as well as monitor treatment response, they emphasize its usefulness for HER2-amplified mCRC, which may benefit patients especially in the first salvage-line treatment [72].

### 3.2. Methylation Status

DNA methylation is a marker that is relatively easy to detect and seems relevant for CRC diagnosis and prognosis (Table 3). Barták et al. noticed that methylated cfDNA fragments are more stable in the circulation, and methylation-related alterations are present in about 65 to 100% of tumor samples (more frequent than mutations: 5–75%) [77]. It was demonstrated that a hypermethylated Septin 9 gene (*SEPT9*) in circulating DNA is a specific CRC biomarker [78,79]. The SEPT9 assay reveals hypermethylation of CpG island 3 in the *SEPT9* promoter [80]. While methylated *SEPT9* levels in tissue and plasma samples are not strongly correlated, methylated *SEPT9* is significantly higher in the plasma of patients with CRC than in patients with no evidence of the disease [81]. 

Hypermethylated *SEPT9* ctDNA disappears after 3 months following surgery, suggesting that this molecule may be the first non-invasive biomarker for postsurgical follow-up [82,83]. The plasma-based *SEPT9* gene methylation assay is currently an FDA-approved non-invasive CRC screening test known as Epi proColon^®^ 1.0 [79,84]. In Europe and some other countries (e.g., China), a second-generation test called Epi proColon^®^ 2.0 CE is available for early-stage CRC screening [85]. The main difference between the generations is that the original Epi proColon algorithm requires only one positive PCR reaction out of two PCRs, emphasizing sensitivity, while the Epi proColon 2.0 CE algorithm requires at least two positive PCR results, placing a greater emphasis on test specificity [86]. In a Chinese opportunistic screening study, a different *SEPT9* gene methylation assay called SensiColon was validated, showing 76.6% specificity and 95.9% sensitivity for the detection of early CRC stages [78]. The overall performance of proposed *SEPT9* gene methylation tests and their comparison with other CRC screening assays have been well reviewed in the study of Song et al. [86]. 

It should be noted that tests based on the methylation status of *SEPT9* may serve as a competitive option for CRC screening and early detection, as it has been demonstrated to have a higher compliance than protein FIT tests and colonoscopy. It could be applied in asymptomatic population screening even if the screening assay does not exhibit sensitivity and specificity equivalent to FIT and FIT-DNA [87], as the uptake rate by the population seems to be a critical aspect of introducing novel screening strategies.

Among the stool-DNA tests, Cologuard™ was the first FDA-approved in vitro diagnostic assay for both left- and right-sided CRCs and pre-malignant neoplasia [85]. Cologuard™ aims to detect 11 distinct biomarkers, classified in three categories: DNA methylation biomarkers in gene promoter regions, such as the specific methylation of N-Myc Downstream-Regulated Gene 4 (*NDRG4*) and Bone Morphogenic Protein (*BMP3*), seven mutational markers in the gene *KRAS*, and the presence of occult hemoglobin. Additionally, the beta-actin gene (*ACTB*) is used as a reference for confirming the total amount of human DNA. In a pivotal case–control study (with colonoscopy as the reference method) involving 10,000 individuals aged 50 to 84 years at average risk, the Cologuard™ DNA test and the FIT (fecal immunochemical test) were used for detecting all CRC stages (I–IV) and showed a sensitivity of 92.3% and 73.8%, respectively [88]. In the detection of CRC stages I–II, DNA testing displays a 70% detection capability [89] and a 42% detection rate of advanced precancerous lesions compared to FIT [88].

**Table 3:** List of methylated cfDNA biomarkers in CRC.

DNA	Source	Function	Technique	Ref.
Methylated *SEPT9*	Plasma	specific non-invasive CRC biomarker for postsurgical follow-up	qPCR	[78,82,83]
CpG island methylation in the INHBB promoter	Serum/stool	biomarker of poor prognosis in CRC	Bisulfite sequencing, qPCR	[90]
Methylation of *APC/MGMT/RASSF2A/Wif-1*	Plasma	biomarker	qPCR	[91]
Methylation of *BMP3/NDRG4/VIM/TFPI2*/mutant *KRAS/ACTB*	Stool	biomarker	QuARTS	[92]

Ref.—reference; ↑—upregulated; ↓—downregulated; MPS—massively parallel sequencing; QuARTS—quantitative allele-specific real-time target and signal amplification.

There are multiple signaling pathways participating in cancer progression, the best studied ones being the mitogen-activated protein kinase (MAPK), p53 and transforming growth factor-beta (TGF-β) pathways. DNA methylation changes affect genes that are involved in these pathways and are considered to be potential biomarkers of CRC. Inhibin subunit beta B (*INHBB*), SPARC related modular calcium binding 2 (*SMOC2*), brain derived neurotrophic factor (*BDNF*), and transforming growth factor beta regulator 4 (*TBRG4*) are highly deregulated by methylation and are involved in CRC metastasis development. Promoter methylation in the above genes is detectable from liquid biopsy samples, but drawbacks of the gold-standard method bisulfite conversion-PCR (cost and labor intensity) currently limit their application in non-invasive screening [93]. In addition to being a diagnostic biomarker, CpG island methylation in the *INHBB* promoter (detected in serum or stool) was reported as a marker of poor prognosis in CRC [90].

Laugsand et al. performed a meta-analysis of available literature sources to find out which methylation-related changes may be used as biomarkers, comparing results from plasma, stool, urine and CRC tissue [94]. The panel *APC/MGMT/RASSF2A/Wif-1* (sensitivity 87%; specificity 92%) appeared to be the most useful in plasma, and *BMP3/NDRG4/VIM/TFPI2*/mutant *KRAS/ACTB* in stool samples.

### 3.3. Genometastasis

There is evidence for the presence of horizontal (cell-to-cell) DNA transfer not only in bacteria, but in mammals as well [95]. The large quantity of tumor-derived cfDNAs in the blood of cancer patients suggests their possible function as carriers of certain “tumorigenic” properties to normal cells [96], supported by the observation that the concentration of cfDNA is five times higher in CRC cases than in healthy individuals [69]. The term “genometastasis” is now used by some authors after some successful experiments on malignized normal cells with tumor-derived cfDNA, such as the transfer of the tumor specific *KRAS* mutation by adding the serum of a CRC patient to a healthy cell line [97]. cfDNA also contains fragments of oncogenes and may behave as an oncovirus, directly participating in metastasis formation [95].

It is worth mentioning that, as CRC carcinogenesis takes several years, some patients may have already donated blood by the time of their diagnosis. As genometastasis may be considered as a possibility, it would be interesting to follow up these donors and their recipients.

### 3.4. mtDNA

The presence of mtDNA in the circulation (cf-mtDNA) opens up new possibilities for the non-invasive analysis of tumor profiles. Several features of the mitochondrial genome can be analyzed, such as mtDNA mutations, mtDNA copy number alterations, heteroplasmy, or cf-mtDNA fragment length distribution. Since mtDNA exhibits characteristics distinct from the nuclear genome, including high copy numbers, high mutation frequencies, and heteroplasmy, it may be considered in some novel applications of liquid biopsies [98]. Recently, some papers have become available on the putative role of cf-mtDNA in CRC screening and follow-up (Table 4).

Haupts et al. reported a higher cf-mtDNA copy number in the plasma of healthy subjects compared to CRC patients [98]. Copy number of mitochondrially encoded NADH ubiquinone oxidoreductase core subunit 1 (*MT-ND1*) in blood plasma has been suggested as a marker of early CRC [102]. Recently, Zhou et al. published results on cf-mtDNA content in urine of CRC patients, finding aberrant fragmentation and mutation profiles with diagnostic potential [101]. mtDNA mutations have been identified in almost every cancer type, including CRC, and are thought to contribute to the development of cancer phenotypes. However, it is not clear if such alterations are the cause or consequence of the changes that take place in tumor cells. Further studies are needed to clarify their role in carcinogenesis.

### 3.5. cfRNA Biomarkers

#### 3.5.1. mRNA

Circulating cell-free mRNA (cf-mRNA) is prone to quick degradation and low abundance [103]. Accordingly, reports of cf-mRNA biomarkers in CRC are relatively scarce in comparison to non-coding RNAs (see below). A loss of glycogen synthase kinase 3 alpha (*GSK3A*) and RAS homolog family member A (*RHOA*) expression in plasma may function as a biomarker of colorectal adenoma, a precancerous lesion of CRC [104]. Another promising transcript from this group seems to be synaptophysin-like 1 (*SYPL1*), detected from stool or plasma samples. This mRNA may potentially be a CRC biomarker rivaling FOBT, carcinoembryonic antigen (CEA) and cancer antigen 19-9 (CA19-9). Moreover, a correlation with tumor size and the clinical stage was observed [105].

Of all CRC patients, approximately 20% are diagnosed with liver metastases at the time of primary tumor diagnosis, and up to 60% develop metachronous metastases [106]. The liver is the most frequent distant location of these metastases and in many cases the only organ affected, being the most common cause of mortality from CRC [107]. The clinical outcome for patients with colorectal liver metastases may be significantly improved by surgical resection. Nevertheless, 50–75% of patients experience recurrence after hepatectomy, most of which occur within 2 years [108,109]. A study by Pun et al. [110] has revealed that plasma levels of the Bmi1 transcript may be used as a biomarker in mCRC patients for non-invasive monitoring of occult metastases and anticipating the emergence of distant metastases. mRNA produced from the genes prostaglandin-endoperoxide synthase 2 (*PTGS2*), guanylate cyclase 2C (*GUCY2C*) and jagged canonical notch ligand 1 (*JAG1*) were reported to be upregulated in mCRC, while the serum expression of *GUCY2C* and *GUCY2C/PTGS2* showed correlation with the therapeutic response [111]. However, a definitive prognostic cf-mRNA biomarker for response to therapy and survival of mCRC patients has yet to emerge.

#### 3.5.2. miRNA

miRNAs are a class of short, non-coding RNAs known to play oncogenic and tumor suppressor roles in various malignancies [112], including CRC, by regulating mRNA targets [113]. They have been reported to circulate in the blood (cf-miRNA), providing diagnostic and prognostic usefulness in oncology [114]. In the past few years, extensive research has been conducted on miRNAs as clinically relevant biomarkers, since miRNAs are present in CRC tumor tissue, feces, and various body fluids, avoiding degradation [115]. The usefulness of various cf-miRNAs as biomarkers is constantly proven from liquid biopsy samples (Table 5).

According to Nassar et al., miRNA panels display better prognostic value than individual miRNAs [117]. Many research groups have reported diagnostic biomarkers for CRC based on blood serum/plasma miRNAs, as they are easy to handle, low-cost, and obtainable with minimal invasiveness. It is worth a note that blood is not the only form of liquid biopsy relevant for CRC management. However, samples such as saliva and urine are still not well studied in large cohorts. In recent years, salivary miRNAs have inspired some growth in interest. Sazanov et al. made measurements of miR-21 obtained from saliva samples of CRC patients. They revealed a significantly increased miR-21 expression compared to healthy individuals with an estimated sensitivity of 97% and specificity of 91% [128].

One of the first large-scale salivary miRNA characterizations in CRC samples has proposed a panel of five miRNAs (miR-186-5p, miR-29a-3p, miR-29c-3p, miR-766-3p, and miR-491-5p) (from 22 miRNAs showing dysregulated patterns), enabling discrimination of CRC patients from healthy controls with a sensitivity of 72% and a specificity of 67% [129]. Urinary miR-129-1-3p and miR-566 have been suggested as biomarkers for early CRC [130]. 

#### 3.5.3. lncRNA

Long non-coding RNAs (lncRNAs) measure over 200 nucleotides in length and are emerging as biomarkers in many cancer types [131]. They are thought to contribute to CRC development and progression by affecting wingless-related integration site/beta-catenin (WNT/β-catenin), phosphatidylinositol 3-kinase/protein kinase B (PI3K/Akt), EGFR, NOTCH, mammalian target of rapamycin (mTOR) and TP53 signaling [132]. They play pivotal roles in the regulation of gene expression in apoptosis and cell proliferation. 

Circulating lncRNA (cf-lncRNA) biomarkers were recently proposed in CRC [133]. Despite their emerging role in cancer, to date, few studies have focused on cf-lncRNA biomarkers in the non-invasive diagnosis and management of CRC patients (Table 6). Other lncRNAs implicated in CRC include plasmacytoma variant translocation 1 (*PVT1*), H19 imprinted maternally expressed transcript (*H19*), metastasis associated lung adenocarcinoma transcript 1 (*MALAT1*), small nucleolar RNA host gene 1, 7 and 15 (*SNHG1, SNHG7, SNHG15*) taurine upregulated 1 (*TUG1*), X inactive specific transcript (*XIST*), regulator of reprogramming (*ROR*) and ZEB-1 antisense RNA 1 (*ZEB1-AS1*) [134].

#### 3.5.4. circRNA

Circular RNA (circRNA) is a type of single-stranded RNA forming a covalently bound loop. Various circRNAs are thought to play important roles in cancer development, affecting processes such as apoptosis, cell-cycle regulation, cell proliferation, migration, and drug resistance development [147,148]. One of the unique features of these RNAs is their covalently closed cyclic structure, making them resistant to digestion by exonucleases. For this reason, their expression in cells is stable, and their half-life is prolonged, particularly in cell-free samples [149]. It has been shown that circRNAs act as miRNA sponges, containing binding sites for miRNA molecules [150]. By this mechanism, circRNAs affect the expression level of various target genes by absorbing miRNAs, participating in the occurrence, development, and progression of CRC (Table 7) [151,152]. 

Hon et al. demonstrated the transfer of drug resistance to sensitive cells via exosomes. They found 105 upregulated and 34 downregulated circRNAs in the FOLFOX-resistant HCT11-R colon cancer cell line. Authors concluded that hsa_circ_0000338 isolated from exosomes could serve as an early predictor of chemoresistance development [166].

Circ-0053277 was shown to sponge miR-2467-3p, promoting proliferation, migration, and epithelial-mesenchymal transition in CRC. Its upregulation was detected in CRC tissue [167], but to our knowledge, it has not been studied yet in liquid biopsies. As implied in Table 7, there are many circRNAs reported as relevant in CRC tissue but not yet detected in the circulation, providing potential targets for future liquid biopsy-based studies. Upregulation of circ_0026416 was reported in plasma, with the authors suggesting that it may act as an oncogene of CRC by sponging miR-136 [168]. Other circRNA biomarkers successfully detected from plasma or serum samples are outlined in Table 8.

### 3.6. Nucleic Acids Released by Gut Microbiota

The human intestinal tract hosts about 1014 bacteria representing more than a thousand species. Intestinal microbiota is generally stable during adult life, but a number of diseases show correlation with altered microbial composition in the gut [174]. Studying the human gut microbiome became very popular recently, with some promising results. Rezasoltani et al. proposed that the most ubiquitous environmental factor in epigenetic modifications may be the gut microbiota [175]. It has been reported that reduced butyrate production, implicated in the development of CRC, may be due to an altered bacterial flora [176]. Establishing the representative pattern of a healthy gut microbiome offers the possibility to associate pattern changes with various diseases.

Components of the gut microbiome release a tremendous amount of nucleic acids and other molecules into the host’s stool and circulation. Studies are emerging to investigate the utility of circulating bacterial DNA (cbDNA) in CRC diagnostics [61]. Some authors have already reported that colorectal neoplasia patients may be distinguished from healthy individuals on this basis [177]. The role of circulating extracellular nucleic acids produced by the gut microbiome is an exciting new direction for research in CRC. However, the difficulty to collect synchronized samples and the lack of standardization protocols represent extra challenges.

## 4. Conclusions

A large number of potential and confirmed cfDNA- and cfRNA-based markers are now available to monitor from peripheral blood, and some even from urine. However, we are still far away from using these biomarkers efficiently in personalized and preventive medical approaches. At this point, liquid biopsy cannot replace a tissue-based histological examination, which remains the essential step to provide basic information on tumor cell properties. Research on the correlation of tumor stage and/or grade with cfNA features is emerging and seems to be the next step for the successful application of liquid biopsy in this context. Additionally, as shown by many of the recent papers we have reviewed, the scientific community still seems to be focused on proposing further novel biomarkers rather than the standardization of methods and quality controls. Despite the availability of a few validated and approved tests such as blood-based Epi proColon and stool-based Cologuard, there is still plenty of potential to improve on these testing strategies in a variety of ways. This is likely to take a lot of time and effort, as new, more efficient methods are still being developed for the detection of low-concentration and fragmented nucleic acids. CRC is a disease that, while deadly in late stages, takes long enough to develop and is often fully curable when caught early. This gives us hope and reason to believe that CRC death tolls may be greatly reduced once cost-efficient liquid biopsy-based methods make the transition into routine medical practice.

## Figures and Tables

**Table 1:** Classification and genetic causes of hereditary CRC syndromes.

Syndrome	Gene(s)	Inheritance	Ref.
Lynch Syndrome (LS)	Heterozygous mutations in MMR genes *MLH1*, *MSH2* (or *EPCAM* deletions), *MSH6* and *PMS2*	AD	[21]
Familial Colorectal Cancer Type X (FCCTX)	*BRCA2*, *SEMA4*, *NTS*, *RASSF9*, *GALNT12*, *KRAS*, *BRAF*, *APC*, *BMPR1A*, and *RPS20*	-	[22,23]
Turcot Syndrome (TS)	MMR genes (*MLH1* and *PMS2*) or *APC*	AD/AR	[24]
Familial Adenomatous Polyposis (FAP)/*APC*-Associated Polyposis	*APC*	AD	[25]
*MUTYH*-Associated Polyposis (MAP)	*MUTYH*	AR	[26]
Polymerase Proofreading-Associated Polyposis (PPAP)	*POLD1*/*POLE*	AD	[27]
NTHL1-Tumor Syndrome	*NTHL1*	AR	[28]
Constitutional MMR Deficiency Syndrome (CMMRD)	Biallelic mutations in MMR genes *MLH1*, *MSH2*, *MSH6* and *PMS2*	AR	[29]
Peutz–Jeghers Syndrome (PJS)	*STK11*/*LKB1*	AD	[30]
*PTEN* Hamartoma Tumor Syndrome (PHTS)	*PTEN*	AD	[31]
Juvenile Polyposis Syndrome (JPS)	*SMAD4*/*BMPR1A*	AD	[32]
Hereditary Mixed Polyposis Syndrome (HMPS)	*GREM1*	AD	[33,34]
RNF43-associated Serrated Polyposis	*RNF43*	AD	[35,36]

Ref.—reference; AD—autosomal dominant; AR—autosomal recessive; MMR—mismatch repair.

**Table 2:** List of cfDNA/ctDNA biomarkers in mutation analysis and CRC management.

Study	Source	Biomarker/Function	Technique	Accuracy
Zhitnyuk et al. [66]	ATAC-seq dataset	cfDNA fragment end profiles/reveal the presence of early-stage colorectal and renal cancers	Anchored multiplex PCR followed by MPS	AUC = 0.94
Cristiano et al. [67]	Plasma	genome-wide fragmentation features/detection of seven cancer types *	Low coverage WGS	AUC = 0.94
Mouliere et al. [68]	Plasma	fragment length and copy number analysis/distinguish cancer from healthy individuals	Low coverage WGS (0.4×)	AUC > 0.99
Flamini et al. [69]	Serum	carriers of certain “tumorigenic” properties	qPCR	AUC = 0.86
Kidess et al. [70]	Plasma and tissue	BRAF, EGFR, KRAS, PIK3CA	SCODA followed by MPS	NA
Vidal et al. [71]	Plasma	RAS/diagnosis and anti-EGFR treatment monitoring of mCRC	OncoBEAM RAS ctDNA assay	NA
Nakamura et al. [72]	Plasma	HER2/monitor anti-HER2 therapy response in mCRC	Guardant360	AUC = 0.53

NA—not available; AUC—area under the ROC curve; MPS—massively parallel sequencing; WGS—whole genome sequencing; * breast, colorectal, lung, ovarian, pancreatic, gastric, and bile duct cancer.

**Table 4:** List of cf-mtDNA copy number changes in CRC.

Study	Source	Exp.	Function	Technique
Haupts et al. [98]	Plasma	↑	potential diagnostic biomarker for CRC screening	MPS
Meddeb et al. [99]	Plasma	↑	diagnostic and prognostic biomarker in metastatic CRC patients	qRT-PCR
Xu et al. [100]	Plasma/tissue	↑	biomarker of early CRC, prediction of tumor response and progression	ddPCR, MPS
Zhou et al. [101]	Urine	↑	monitoring of aberrant fragmentation and mutation profiles	MPS

Exp.—expression; ↑—upregulated; MPS—massively parallel sequencing; ddPCR—droplet digital PCR.

**Table 5:** List of cf-miRNAs and their role in CRC. Several miRNAs have been present in EMVs that may be extracted from a serum/plasma source.

cf-miRNAs	Source	Exp.	Targets	Biomarker/Function	Ref.
miR-1290	Plasma	↑	epithelial-mesenchymal transition (EMT) markers	prognostic/poor overall survival, advanced TNM stage	[116]
miR-21 miR-145 miR-203 miR-155 miR-210 miR-31 miR-345	Plasma	↑	various downstream targets (e.g., *PTEN*, *PDCD4* genes; WNT/β-Catenin signaling pathway, etc.)	diagnostic/differentiation of surgery- naïve CRC patients, diagnosis of liver metastases	[117]
miR-92a	Plasma/ EMVs	↑	signaling pathways-BMPs/SMAD; WNT/β-Catenin; *PTEN*/*AKT*/*FoxO;* genes-*DKK3, KLF4, SMAD7*	diagnostic/distinguishing advanced neoplasia CRC patients, early CRC screening	[118,119]
miR-92b	Plasma/ EMVs	↓	NA	diagnostic/early CRC detection	[120]
miR-17-5p mmiR-92a-3p	Serum/ EMVs	↑	NA	prognostic/primary and mCRC diagnosis, correlation with stages and grades of CRC	[121]
miR-150-5p	Serum/ EMVs	↓	*ZEB1*	diagnostic and prognostic/poor differentiation, positive lymph node metastasis, TNM stage	[122]
miR-122	Serum/ EMVs	↑	*PKM2*	prognostic/differentiation of CRC patients with liver metastasis	[123]
miR-1290	Serum	↑	various tumor suppressors (e.g., Forkhead box protein-A1, N-acetyltransferase etc.)	diagnostic/early CRC detection, recurrence monitoring, tumor aggressivity	[124]
miR-30e-3p, mmiR-146a-5p/ mmiR-148a-3p	Serum	↑|↓	miR-146a-5p via carboxypeptidase M/src-FAK	diagnostic	[125]
miR-1247-5p miR-1293 miR-548at-5p miR-107 miR-139-3p	Serum	↓	various downstream targets	diagnostic/detection of precancerous polyps and early CRC stages	[126]
miR-19a miR-20a miR-150 let-7a | miR-143 miR-145	Serum	↑|↓	various downstream targets	diagnostic, prognostic/CRC screening, TNM staging and LNM status determination	[127]
miR-21	Plasma/saliva	↑	MAPK, WNT/β-Catenin signaling/*PTEN*, *PDCD*, *DKK2*	diagnostic and prognostic/CRC screening	[128]
miR-186-5p miR-29a-3p miR-29c-3p miR-766-3p miR-491-5p	Saliva	↑	various downstream targets	diagnostic and prognostic/distinguishing CRC from healthy controls, predicting disease outcome in advanced stages	[129]
miR-129-1-3p mmiR-566	Urine	↑	NA	diagnostic/early CRC detection	[130]

Exp.—expression; Ref.—references; ↑—upregulated; ↓—downregulated; NA—not available; EMVs—extracellular membrane vesicles.

**Table 6:** List of cf-lncRNAs and their role in CRC. Several lncRNAs are present in EMVs that may be extracted from serum/plasma samples.

cf-lncRNAs	Source	Exp.	Biomarker/Function	Ref.
NEAT1 variant 1/variant 2	Whole blood	↑	diagnostic	[135]
BLACAT1	Serum	↑	diagnostic/distinguishing CRC patients, non-cancer patients and healthy individuals	[136]
CCAT2, HULC	Serum	↑	diagnostic/screening of CRC or adenomatous polyps	[137]
CCAT2	Serum/EMVs	↑	diagnostic	[138]
CRNDE-h	Serum/EMVs	↑	prognostic and diagnostic/low overall survival of CRC patients	[139]
UCA1	Serum/EMVs	↑	predictive/resistance to cetuximab	[140]
FOXD2-AS1 NRIR XLOC_0009459	EMVs	↑	diagnostic/early-stage CRC diagnosis	[141]
ATB, CCAT1	Plasma	↑	diagnostic	[142]
CCAT1, HOTAIR|p21	Plasma	↑|↓	prognostic	[143]
HOTAIR	Plasma	↓	prognostic/increase radiosensitivity via miRNA-93/ATG12 axis	[144]
LNCV6_116109/98390/38772/108266/84003/98602	Plasma/EMVs	↑	diagnostic/early-stage CRC diagnosis	[145]
TCONS_00026334	NA	↓	tumor suppressor/suppress CRC progression via miR-548n/TP53ONP1 axis	[146]

Exp.—expression; Ref.—references; ↑—upregulated; ↓—downregulated; EMVs—extracellular membrane vesicles.

**Table 7:** List of circRNAs relevant for CRC progression and clinical/biological features (not all have been studied in liquid biopsies).

circRNA	Target Molecules/Genes	Function	Ref.
hsa-circ-000984	miR-106b/↑ of *CDK6*	proliferation, metastasis	[153]
hsa-circ-0005927	miR-942-5p/↓ of *BATF2*	cell colony-forming ability, apoptosis, migration	[154]
circPACRGL	miR-142-3p, miR-506-3p/↑ of *TGF-β1*	proliferation, metastasis (migration and invasion)	[155]
hsa-circ-0009361	miR-582-3p/↓ of *APC2*/WNT/β-catenin signaling path	suppress cell growth and metastasis	[156]
circCCDC66	various oncogenes	proliferation, migration, invasion	[157]
circ-FBXW7	*NEK2*, *mTOR*, *PTEN*	proliferation, migration, invasion	[158]
hsa-circ-0001178	miR-382/587/616/↑ of *ZEB1*	metastasis, invasion	[159]
hsa-circ-DDX17	miR-31-5p/↓ of *KANK1*	promotes sensitivity to 5-FU	[160]
ciRS-122	miR-122/↑ of *PKM2*	promotes resistance to oxaliplatin	[161]
hsa-circ-001680	miR-340/↑ of *BMI1*	promotes chemoresistance to irinotecan	[162]
circ-FBXW7	miR-18b-5p	ameliorates chemoresistance to oxaliplatin	[163]
circ-CSPP1	miR-944/↓ of FZD7	enhanced doxorubicin sensitivity	[164]
circ-0000338	miR-217, miR-485-3p	enhanced 5-FU resistance	[165]

Ref.—references; ↑—upregulated; ↓—downregulated.

**Table 8:** List of circRNAs described as biomarkers for CRC diagnosis and screening (from liquid biopsies).

circRNA	Source	Exp.	Biomarker/Function	Ref.
hsa-circ-0006282	Plasma	↑	diagnostic; improving the detection and monitoring of CRCs in combination with carcinoembryonic antigen (CEA) and carbohydrate antigen199 (CA199)	[169]
hsa-circ-0001900 hsa-circ-0001178 hsa-circ-0005927	Plasma	↑	diagnostic/improving the detection of CAE-negative CRC	[170]
hsa-circ-0082182 hsa-circ-0000370| hsa-circ-0035455	Plasma	↑|↓	diagnostic	[171]
hsa-circ-0004771	Serum/ EMVs	↑	diagnostic/differentiation of benign intestinal diseases, stage I/II CRCs, and CRCs from healthy individuals	[172]
circ-FMN2 circ-LMNB1 circ-ZNF609	Serum	↑	diagnostic and prognostic/correlation with histopathological grade, lymph node metastasis, TNM stages	[173]

Ref.—references; ↑—upregulated; ↓—downregulated; EMVs—extracellular membrane vesicles.

## References

[1] Currais P., Rosa I., Claro I. (2022). Colorectal Cancer Carcinogenesis: From Bench to Bedside. World J. Gastrointest. Oncol..

[2] Sung H., Ferlay J., Siegel R.L., Laversanne M., Soerjomataram I., Jemal A., Bray F. (2021). Global Cancer Statistics 2020: GLOBOCAN Estimates of Incidence and Mortality Worldwide for 36 Cancers in 185 Countries. CA Cancer J. Clin..

[3] Wong H.H., Chu P. (2012). Immunohistochemical Features of the Gastrointestinal Tract Tumors. J. Gastrointest. Oncol..

[4] Reddy R.M., Fleshman J.W. (2006). Colorectal Gastrointestinal Stromal Tumors: A Brief Review. Clin. Colon Rectal Surg..

[5] Alyabsi M., Sabatin F., Ramadan M., Jazieh A.R. (2021). Colorectal Cancer Survival among Ministry of National Guard-Health Affairs (MNG-HA) Population 2009-2017: Retrospective Study. BMC Cancer.

[6] Lansdorp-Vogelaar I., van Ballegooijen M., Zauber A.G., Habbema J.D.F., Kuipers E.J. (2009). Effect of Rising Chemotherapy Costs on the Cost Savings of Colorectal Cancer Screening. J. Natl. Cancer Inst..

[7] Siegel R.L., Miller K.D., Jemal A. (2018). Cancer Statistics, 2018. CA Cancer J. Clin..

[8] Latchford A. (2020). How Should Colonoscopy Surveillance in Lynch Syndrome Be Performed?. Gastroenterology.

[9] Delcò F., Sonnenberg A. (1999). Limitations of the Faecal Occult Blood Test in Screening for Colorectal Cancer. Ital. J. Gastroenterol. Hepatol..

[10] Lee J.K., Reis V., Liu S., Conn L., Groessl E.J., Ganiats T.G., Ho S.B. (2009). Improving Fecal Occult Blood Testing Compliance Using a Mailed Educational Reminder. J. Gen. Intern. Med..

[11] Bretthauer M., Kaminski M.F., Løberg M., Zauber A.G., Regula J., Kuipers E.J., Hernán M.A., McFadden E., Sunde A., Kalager M. (2016). Population-Based Colonoscopy Screening for Colorectal Cancer: A Randomized Clinical Trial. JAMA Intern. Med..

[12] Dhaliwal A., Vlachostergios P.J., Oikonomou K.G., Moshenyat Y. (2015). Fecal DNA Testing for Colorectal Cancer Screening: Molecular Targets and Perspectives. World J. Gastrointest. Oncol..

[13] Fan C.-W., Kuo Y.-B., Lin G.-P., Chen S.-M., Chang S.-H., Li B.-A., Chan E.-C. (2017). Development of a Multiplexed Tumor-Associated Autoantibody-Based Blood Test for the Detection of Colorectal Cancer. Clin. Chim. Acta.

[14] Ladabaum U., Allen J., Wandell M., Ramsey S. (2013). Colorectal Cancer Screening with Blood-Based Biomarkers: Cost-Effectiveness of Methylated Septin 9 DNA versus Current Strategies. Cancer Epidemiol. Biomarkers Prev..

[15] Ross J.S., Fakih M., Ali S.M., Elvin J.A., Schrock A.B., Suh J., Vergilio J.-A., Ramkissoon S., Severson E., Daniel S. (2018). Targeting HER2 in Colorectal Cancer: The Landscape of Amplification and Short Variant Mutations in ERBB2 and ERBB3. Cancer.

[16] Sameer A.S. (2013). Colorectal Cancer: Molecular Mutations and Polymorphisms. Front. Oncol..

[17] Nojadeh J.N., Behrouz Sharif S., Sakhinia E. (2018). Microsatellite Instability in Colorectal Cancer. EXCLI J..

[18] Arvelo F., Sojo F., Cotte C. (2015). Biology of Colorectal Cancer. Ecancermedicalscience.

[19] Lin O.S. (2012). Colorectal Cancer Screening in Patients at Moderately Increased Risk due to Family History. World J. Gastrointest. Oncol..

[20] Daca Alvarez M., Quintana I., Terradas M., Mur P., Balaguer F., Valle L. (2021). The Inherited and Familial Component of Early-Onset Colorectal Cancer. Cells.

[21] Buglyó G., Styk J., Pös O., Csók Á., Repiska V., Soltész B., Szemes T., Nagy B. (2022). Liquid Biopsy as a Source of Nucleic Acid Biomarkers in the Diagnosis and Management of Lynch Syndrome. Int. J. Mol. Sci..

[22] Nejadtaghi M., Jafari H., Farrokhi E., Samani K.G. (2017). Familial Colorectal Cancer Type X (FCCTX) and the Correlation with Various Genes-A Systematic Review. Curr. Probl. Cancer.

[23] Nieminen T.T., O’Donohue M.-F., Wu Y., Lohi H., Scherer S.W., Paterson A.D., Ellonen P., Abdel-Rahman W.M., Valo S., Mecklin J.-P. (2014). Germline Mutation of RPS20, Encoding a Ribosomal Protein, Causes Predisposition to Hereditary Nonpolyposis Colorectal Carcinoma without DNA Mismatch Repair Deficiency. Gastroenterology.

[24] Khattab A., Monga D.K. (2021). Turcot Syndrome. StatPearls [Internet].

[25] Nielsen M., Aretz S. (2018). Familial Adenomatous Polyposis or APC-Associated Polyposis. Hered. Colorectal Cancer.

[26] Magrin L., Fanale D., Brando C., Corsini L.R., Randazzo U., Di Piazza M., Gurrera V., Pedone E., Bazan Russo T.D., Vieni S. (2022). MUTYH-Associated Tumor Syndrome: The Other Face of MAP. Oncogene.

[27] Palles C., Latchford A., Valle L. (2018). Adenomatous Polyposis Syndromes: Polymerase Proofreading-Associated Polyposis. Hered. Colorectal Cancer.

[28] Weren R.D.A., Ligtenberg M.J.L., Kets C.M., de Voer R.M., Verwiel E.T.P., Spruijt L., van Zelst-Stams W.A.G., Jongmans M.C., Gilissen C., Hehir-Kwa J.Y. (2015). A Germline Homozygous Mutation in the Base-Excision Repair Gene NTHL1 Causes Adenomatous Polyposis and Colorectal Cancer. Nat. Genet..

[29] Wimmer K., Kratz C.P., Vasen H.F.A., Caron O., Colas C., Entz-Werle N., Gerdes A.-M., Goldberg Y., Ilencikova D., Muleris M. (2014). Diagnostic Criteria for Constitutional Mismatch Repair Deficiency Syndrome: Suggestions of the European Consortium “Care for CMMRD” (C4CMMRD). J. Med. Genet..

[30] Wagner A., Aretz S., Auranen A., Bruno M.J., Cavestro G.M., Crosbie E.J., Goverde A., Jelsig A.M., Latchford A., van Leerdam M.E. (2021). The Management of Peutz-Jeghers Syndrome: European Hereditary Tumour Group (EHTG) Guideline. J. Clin. Med. Res..

[31] Pilarski R. (2019). Hamartoma Tumor Syndrome: A Clinical Overview. Cancers.

[32] Dal Buono A., Gaiani F., Poliani L., Laghi L. (2022). Juvenile Polyposis Syndrome: An Overview. Best Pract. Res. Clin. Gastroenterol..

[33] Lieberman S., Walsh T., Schechter M., Adar T., Goldin E., Beeri R., Sharon N., Baris H., Ben Avi L., Half E. (2017). Features of Patients With Hereditary Mixed Polyposis Syndrome Caused by Duplication of GREM1 and Implications for Screening and Surveillance. Gastroenterology.

[34] Ballester-Vargas V., Tomlinson I. (2016). Hereditary Mixed Polyposis Syndrome. Intest. Polyposis Syndr..

[35] Taupin D., Lam W., Rangiah D., McCallum L., Whittle B., Zhang Y., Andrews D., Field M., Goodnow C.C., Cook M.C. (2015). A Deleterious RNF43 Germline Mutation in a Severely Affected Serrated Polyposis Kindred. Hum. Genome Var..

[36] Quintana I., Mejías-Luque R., Terradas M., Navarro M., Piñol V., Mur P., Belhadj S., Grau E., Darder E., Solanes A. (2018). Evidence Suggests That Germline Mutations Are a Rare Cause of Serrated Polyposis. Gut.

[37] Yurgelun M.B., Kulke M.H., Fuchs C.S., Allen B.A., Uno H., Hornick J.L., Ukaegbu C.I., Brais L.K., McNamara P.G., Mayer R.J. (2017). Cancer Susceptibility Gene Mutations in Individuals With Colorectal Cancer. J. Clin. Oncol..

[38] Tutlewska K., Lubinski J., Kurzawski G. (2013). Germline Deletions in the EPCAM Gene as a Cause of Lynch Syndrome—Literature Review. Hered. Cancer Clin. Pract..

[39] Lynch H.T., Lynch P.M., Lanspa S.J., Snyder C.L., Lynch J.F., Boland C.R. (2009). Review of the Lynch Syndrome: History, Molecular Genetics, Screening, Differential Diagnosis, and Medicolegal Ramifications. Clin. Genet..

[40] Lynch H.T., Shaw T.G. (2013). Practical Genetics of Colorectal Cancer. Chin. Clin. Oncol..

[41] Balaguer F., Moreira L., Lozano J.J., Link A., Ramirez G., Shen Y., Cuatrecasas M., Arnold M., Meltzer S.J., Syngal S. (2011). Colorectal Cancers with Microsatellite Instability Display Unique miRNA Profiles. Clin. Cancer Res..

[42] Giardiello F.M., Trimbath J.D. (2006). Peutz-Jeghers Syndrome and Management Recommendations. Clin. Gastroenterol. Hepatol..

[43] Chae H.-D., Jeon C.-H. (2014). Peutz-Jeghers Syndrome with Germline Mutation of STK11. Ann. Surg. Treat. Res..

[44] Goodenberger M., Lindor N.M. (2011). Lynch Syndrome and MYH-Associated Polyposis: Review and Testing Strategy. J. Clin. Gastroenterol..

[45] Bogaert J., Prenen H. (2014). Molecular Genetics of Colorectal Cancer. Ann. Gastroenterol. Hepatol..

[46] Sweetser S., Smyrk T.C., Sinicrope F.A. (2013). Serrated Colon Polyps as Precursors to Colorectal Cancer. Clin. Gastroenterol. Hepatol..

[47] Gala M.K., Mizukami Y., Le L.P., Moriichi K., Austin T., Yamamoto M., Lauwers G.Y., Bardeesy N., Chung D.C. (2014). Germline Mutations in Oncogene-Induced Senescence Pathways Are Associated with Multiple Sessile Serrated Adenomas. Gastroenterology.

[48] Le D.T., Durham J.N., Smith K.N., Wang H., Bartlett B.R., Aulakh L.K., Lu S., Kemberling H., Wilt C., Luber B.S. (2017). Mismatch Repair Deficiency Predicts Response of Solid Tumors to PD-1 Blockade. Science.

[49] Rudolph K.L., Millard M., Bosenberg M.W., DePinho R.A. (2001). Telomere Dysfunction and Evolution of Intestinal Carcinoma in Mice and Humans. Nat. Genet..

[50] Ayiomamitis G.D., Notas G., Zaravinos A., Zizi-Sermpetzoglou A., Georgiadou M., Sfakianaki O., Kouroumallis E. (2014). Differences in Telomerase Activity between Colon and Rectal Cancer. Can. J. Surg..

[51] Wang J.-Y., Hsieh J.-S., Chang M.-Y., Huang T.-J., Chen F.-M., Cheng T.-L., Alexandersen K., Huang Y.-S., Tzou W.-S., Lin S.-R. (2004). Molecular Detection of APC, K-Ras, and p53 Mutations in the Serum of Colorectal Cancer Patients as Circulating Biomarkers. World J. Surg..

[52] Midthun L., Shaheen S., Deisch J., Senthil M., Tsai J., Hsueh C.-T. (2019). Concomitant and Mutations in Colorectal Cancer. J. Gastrointest. Oncol..

[53] Mauri G., Vitiello P.P., Sogari A., Crisafulli G., Sartore-Bianchi A., Marsoni S., Siena S., Bardelli A. (2022). Liquid Biopsies to Monitor and Direct Cancer Treatment in Colorectal Cancer. Br. J. Cancer.

[54] Zygulska A.L., Pierzchalski P. (2022). Novel Diagnostic Biomarkers in Colorectal Cancer. Int. J. Mol. Sci..

[55] Umwali Y., Yue C.-B., Gabriel A.N.A., Zhang Y., Zhang X. (2021). Roles of Exosomes in Diagnosis and Treatment of Colorectal Cancer. World J. Clin. Cases.

[56] Ruiz-López L., Blancas I., Garrido J.M., Mut-Salud N., Moya-Jódar M., Osuna A., Rodríguez-Serrano F. (2018). The Role of Exosomes on Colorectal Cancer: A Review. J. Gastroenterol. Hepatol..

[57] Pös O., Biró O., Szemes T., Nagy B. (2018). Circulating Cell-Free Nucleic Acids: Characteristics and Applications. Eur. J. Hum. Genet..

[58] Szilágyi M., Pös O., Márton É., Buglyó G., Soltész B., Keserű J., Penyige A., Szemes T., Nagy B. (2020). Circulating Cell-Free Nucleic Acids: Main Characteristics and Clinical Application. Int. J. Mol. Sci..

[59] Aucamp J., Bronkhorst A.J., Badenhorst C.P.S., Pretorius P.J. (2018). The Diverse Origins of Circulating Cell-Free DNA in the Human Body: A Critical Re-Evaluation of the Literature. Biol. Rev. Camb. Philos. Soc..

[60] Hu Z., Chen H., Long Y., Li P., Gu Y. (2021). The Main Sources of Circulating Cell-Free DNA: Apoptosis, Necrosis and Active Secretion. Crit. Rev. Oncol. Hematol..

[61] Glyn T., Purcell R. (2022). Circulating Bacterial DNA: A New Paradigm for Cancer Diagnostics. Front. Med..

[62] Dasari A., Morris V.K., Allegra C.J., Atreya C., Benson A.B., Boland P., Chung K., Copur M.S., Corcoran R.B., Deming D.A. (2020). ctDNA Applications and Integration in Colorectal Cancer: An NCI Colon and Rectal-Anal Task Forces Whitepaper. Nat. Rev. Clin. Oncol..

[63] Frattini M., Gallino G., Signoroni S., Balestra D., Lusa L., Battaglia L., Sozzi G., Bertario L., Leo E., Pilotti S. (2008). Quantitative and Qualitative Characterization of Plasma DNA Identifies Primary and Recurrent Colorectal Cancer. Cancer Lett..

[64] Cassinotti E., Boni L., Segato S., Rausei S., Marzorati A., Rovera F., Dionigi G., David G., Mangano A., Sambucci D. (2013). Free Circulating DNA as a Biomarker of Colorectal Cancer. Int. J. Surg..

[65] Tie J., Cohen J.D., Lahouel K., Lo S.N., Wang Y., Kosmider S., Wong R., Shapiro J., Lee M., Harris S. (2022). Circulating Tumor DNA Analysis Guiding Adjuvant Therapy in Stage II Colon Cancer. N. Engl. J. Med..

[66] Zhitnyuk Y.V., Koval A.P., Alferov A.A., Shtykova Y.A., Mamedov I.Z., Kushlinskii N.E., Chudakov D.M., Shcherbo D.S. (2022). Deep cfDNA Fragment End Profiling Enables Cancer Detection. Mol. Cancer.

[67] Cristiano S., Leal A., Phallen J., Fiksel J., Adleff V., Bruhm D.C., Jensen S.Ø., Medina J.E., Hruban C., White J.R. (2019). Genome-Wide Cell-Free DNA Fragmentation in Patients with Cancer. Nature.

[68] Mouliere F., Chandrananda D., Piskorz A.M., Moore E.K., Morris J., Ahlborn L.B., Mair R., Goranova T., Marass F., Heider K. (2018). Enhanced Detection of Circulating Tumor DNA by Fragment Size Analysis. Sci. Transl. Med..

[69] Flamini E., Mercatali L., Nanni O., Calistri D., Nunziatini R., Zoli W., Rosetti P., Gardini N., Lattuneddu A., Verdecchia G.M. (2006). Free DNA and Carcinoembryonic Antigen Serum Levels: An Important Combination for Diagnosis of Colorectal Cancer. Clin. Cancer Res..

[70] Kidess E., Heirich K., Wiggin M., Vysotskaia V., Visser B.C., Marziali A., Wiedenmann B., Norton J.A., Lee M., Jeffrey S.S. (2015). Mutation Profiling of Tumor DNA from Plasma and Tumor Tissue of Colorectal Cancer Patients with a Novel, High-Sensitivity Multiplexed Mutation Detection Platform. Oncotarget.

[71] Vidal J., Muinelo L., Dalmases A., Jones F., Edelstein D., Iglesias M., Orrillo M., Abalo A., Rodríguez C., Brozos E. (2017). Plasma ctDNA RAS Mutation Analysis for the Diagnosis and Treatment Monitoring of Metastatic Colorectal Cancer Patients. Ann. Oncol..

[72] Nakamura Y., Okamoto W., Kato T., Esaki T., Kato K., Komatsu Y., Yuki S., Masuishi T., Nishina T., Ebi H. (2021). Circulating Tumor DNA-Guided Treatment with Pertuzumab plus Trastuzumab for HER2-Amplified Metastatic Colorectal Cancer: A Phase 2 Trial. Nat. Med..

[73] Mouliere F., Robert B., Arnau Peyrotte E., Del Rio M., Ychou M., Molina F., Gongora C., Thierry A.R. (2011). High Fragmentation Characterizes Tumour-Derived Circulating DNA. PLoS ONE.

[74] Henriksen T.V., Reinert T., Christensen E., Sethi H., Birkenkamp-Demtröder K., Gögenur M., Gögenur I., Zimmermann B.G., Dyrskjøt L., IMPROVE Study Group (2020). The Effect of Surgical Trauma on Circulating Free DNA Levels in Cancer Patients-Implications for Studies of Circulating Tumor DNA. Mol. Oncol..

[75] Osumi H., Shinozaki E., Yamaguchi K., Zembutsu H. (2019). Clinical Utility of Circulating Tumor DNA for Colorectal Cancer. Cancer Sci..

[76] Ahcene Djaballah S., Daniel F., Milani A., Ricagno G., Lonardi S. (2022). HER2 in Colorectal Cancer: The Long and Winding Road From Negative Predictive Factor to Positive Actionable Target. Am. Soc. Clin. Oncol. Educ. Book.

[77] Barták B.K., Nagy Z.B., Spisák S., Tulassay Z., Dank M., Igaz P., Molnár B. (2018). In vivo analysis of circulating cell-free DNA release and degradation. Orv. Hetil..

[78] Wu D., Zhou G., Jin P., Zhu J., Li S., Wu Q., Wang G., Sheng J., Wang J., Song L. (2016). Detection of Colorectal Cancer Using a Simplified SEPT9 Gene Methylation Assay Is a Reliable Method for Opportunistic Screening. J. Mol. Diagn..

[79] Song L., Li Y., Jia J., Zhou G., Wang J., Kang Q., Jin P., Sheng J., Cai G., Cai S. (2016). Algorithm Optimization in Methylation Detection with Multiple RT-qPCR. PLoS ONE.

[80] Wasserkort R., Kalmar A., Valcz G., Spisak S., Krispin M., Toth K., Tulassay Z., Sledziewski A.Z., Molnar B. (2013). Aberrant Septin 9 DNA Methylation in Colorectal Cancer Is Restricted to a Single CpG Island. BMC Cancer.

[81] Tóth K., Wasserkort R., Sipos F., Kalmár A., Wichmann B., Leiszter K., Valcz G., Juhász M., Miheller P., Patai Á.V. (2014). Detection of Methylated Septin 9 in Tissue and Plasma of Colorectal Patients with Neoplasia and the Relationship to the Amount of Circulating Cell-Free DNA. PLoS ONE.

[82] Leon Arellano M., García-Arranz M., Ruiz R., Olivera R., Magallares S., Olmedillas-Lopez S., Valdes-Sanchez T., Guadalajara H., García-Olmo D. (2020). A First Step to a Biomarker of Curative Surgery in Colorectal Cancer by Liquid Biopsy of Methylated Septin 9 Gene. Dis. Markers.

[83] Wills B., Gorse E., Lee V. (2018). Role of Liquid Biopsies in Colorectal Cancer. Curr. Probl. Cancer.

[84] Provenzale D., Gupta S., Ahnen D.J., Markowitz A.J., Chung D.C., Mayer R.J., Regenbogen S.E., Blanco A.M., Bray T., Cooper G. (2018). NCCN Guidelines Insights: Colorectal Cancer Screening, Version 1.2018. J. Natl. Compr. Cancer Netw..

[85] Lamb Y.N., Dhillon S. (2017). Epi proColon 2.0 CE: A Blood-Based Screening Test for Colorectal Cancer. Mol. Diagn. Ther..

[86] Song L., Jia J., Peng X., Xiao W., Li Y. (2017). The Performance of the SEPT9 Gene Methylation Assay and a Comparison with Other CRC Screening Tests: A Meta-Analysis. Sci. Rep..

[87] Adler A., Geiger S., Keil A., Bias H., Schatz P., deVos T., Dhein J., Zimmermann M., Tauber R., Wiedenmann B. (2014). Improving Compliance to Colorectal Cancer Screening Using Blood and Stool Based Tests in Patients Refusing Screening Colonoscopy in Germany. BMC Gastroenterol..

[88] Imperiale T.F., Ransohoff D.F., Itzkowitz S.H., Levin T.R., Lavin P., Lidgard G.P., Ahlquist D.A., Berger B.M. (2014). Multitarget Stool DNA Testing for Colorectal-Cancer Screening. N. Engl. J. Med..

[89] Ahlquist D.A. (2015). Multi-Target Stool DNA Test: A New High Bar for Noninvasive Screening. Dig. Dis. Sci..

[90] Mayor R., Casadomé L., Azuara D., Moreno V., Clark S.J., Capellà G., Peinado M.A. (2009). Long-Range Epigenetic Silencing at 2q14.2 Affects Most Human Colorectal Cancers and May Have Application as a Non-Invasive Biomarker of Disease. Br. J. Cancer.

[91] Lee B.B., Lee E.J., Jung E.H., Chun H.-K., Chang D.K., Song S.Y., Park J., Kim D.-H. (2009). Aberrant Methylation of APC, MGMT, RASSF2A, and Wif-1 Genes in Plasma as a Biomarker for Early Detection of Colorectal Cancer. Clin. Cancer Res..

[92] Ahlquist D.A., Taylor W.R., Mahoney D.W., Zou H., Domanico M., Thibodeau S.N., Boardman L.A., Berger B.M., Lidgard G.P. (2012). The Stool DNA Test Is More Accurate than the Plasma Septin 9 Test in Detecting Colorectal Neoplasia. Clin. Gastroenterol. Hepatol..

[93] Gutierrez A., Demond H., Brebi P., Ili C.G. (2021). Novel Methylation Biomarkers for Colorectal Cancer Prognosis. Biomolecules.

[94] Laugsand E.A., Brenne S.S., Skorpen F. (2021). DNA Methylation Markers Detected in Blood, Stool, Urine, and Tissue in Colorectal Cancer: A Systematic Review of Paired Samples. Int. J. Colorectal Dis..

[95] Trejo-Becerril C., Pérez-Cárdenas E., Taja-Chayeb L., Anker P., Herrera-Goepfert R., Medina-Velázquez L.A., Hidalgo-Miranda A., Pérez-Montiel D., Chávez-Blanco A., Cruz-Velázquez J. (2012). Cancer Progression Mediated by Horizontal Gene Transfer in an in Vivo Model. PLoS ONE.

[96] Alekseeva L., Mironova N. (2021). Role of Cell-Free DNA and Deoxyribonucleases in Tumor Progression. Int. J. Mol. Sci..

[97] Thierry A.R., El Messaoudi S., Gahan P.B., Anker P., Stroun M. (2016). Origins, Structures, and Functions of Circulating DNA in Oncology. Cancer Metastasis Rev..

[98] Haupts A., Vogel A., Foersch S., Hartmann M., Maderer A., Wachter N., Huber T., Kneist W., Roth W., Lang H. (2021). Comparative Analysis of Nuclear and Mitochondrial DNA from Tissue and Liquid Biopsies of Colorectal Cancer Patients. Sci. Rep..

[99] Meddeb R., Dache Z.A.A., Thezenas S., Otandault A., Tanos R., Pastor B., Sanchez C., Azzi J., Tousch G., Azan S. (2019). Quantifying Circulating Cell-Free DNA in Humans. Sci. Rep..

[100] Xu Y., Zhou J., Yuan Q., Su J., Li Q., Lu X., Zhang L., Cai Z., Han J. (2021). Quantitative Detection of Circulating MT-ND1 as a Potential Biomarker for Colorectal Cancer. Bosn. J. Basic Med. Sci..

[101] Zhou K., Liu Y., Yuan Q., Lai D., Guo S., Wang Z., Su L., Zhang H., Wang X., Guo W. (2022). Next-Generation Sequencing-Based Analysis of Urine Cell-Free mtDNA Reveals Aberrant Fragmentation and Mutation Profile in Cancer Patients. Clin. Chem..

[102] Thyagarajan B., Guan W., Fedirko V., Barcelo H., Tu H., Gross M., Goodman M., Bostick R.M. (2016). No Association between Mitochondrial DNA Copy Number and Colorectal Adenomas. Mol. Carcinog..

[103] Rapisuwon S., Vietsch E.E., Wellstein A. (2016). Circulating Biomarkers to Monitor Cancer Progression and Treatment. Comput. Struct. Biotechnol. J..

[104] Xue V.W., Cheung M.T., Chan P.T., Luk L.L.Y., Lee V.H., Au T.C., Yu A.C., Cho W.C.S., Tsang H.F.A., Chan A.K. (2019). Non-Invasive Potential Circulating mRNA Markers for Colorectal Adenoma Using Targeted Sequencing. Sci. Rep..

[105] Shu T., Wu K., Guo Y., He Q., Song X., Shan J., Wu L., Liu J., Wang Z., Liu L. (2022). Evaluation of Fecal SYPL1 as a Diagnostic Biomarker in Colorectal Cancer. Clin. Biochem..

[106] Engstrand J., Nilsson H., Strömberg C., Jonas E., Freedman J. (2018). Colorectal Cancer Liver Metastases—A Population-Based Study on Incidence, Management and Survival. BMC Cancer.

[107] Favoriti P., Carbone G., Greco M., Pirozzi F., Pirozzi R.E.M., Corcione F. (2016). Worldwide Burden of Colorectal Cancer: A Review. Updates Surg..

[108] Battula N., Tsapralis D., Mayer D., Isaac J., Muiesan P., Sutcliffe R.P., Bramhall S., Mirza D., Marudanayagam R. (2014). Repeat Liver Resection for Recurrent Colorectal Metastases: A Single-Centre, 13-Year Experience. HPB.

[109] Imai K., Allard M.-A., Benitez C.C., Vibert E., Sa Cunha A., Cherqui D., Castaing D., Bismuth H., Baba H., Adam R. (2016). Early Recurrence After Hepatectomy for Colorectal Liver Metastases: What Optimal Definition and What Predictive Factors?. Oncologist.

[110] Pun J.C.-S., Chan J.Y.-J., Chun B.K.-M., Ng K.-W., Tsui S.Y.-K., Wan T.M.-H., Lo O., Poon J.T.-C., Ng L., Pang R. (2014). Plasma Bmi1 mRNA as a Potential Prognostic Biomarker for Distant Metastasis in Colorectal Cancer Patients. Mol. Clin. Oncol..

[111] Jimenez-Luna C., González-Flores E., Ortiz R., Martínez-González L.J., Antúnez-Rodríguez A., Expósito-Ruiz M., Melguizo C., Caba O., Prados J. (2021). Circulating *PTGS2*, *JAG1*, *GUCY2C* and *PGF* mRNA in Peripheral Blood and Serum as Potential Biomarkers for Patients with Metastatic Colon Cancer. J. Clin. Med. Res..

[112] Kong Y.W., Ferland-McCollough D., Jackson T.J., Bushell M. (2012). microRNAs in Cancer Management. Lancet Oncol..

[113] Peng Y., Croce C.M. (2016). The Role of MicroRNAs in Human Cancer. Signal Transduct. Target. Ther..

[114] Sohel M.M.H. (2020). Circulating microRNAs as Biomarkers in Cancer Diagnosis. Life Sci..

[115] Turchinovich A., Weiz L., Langheinz A., Burwinkel B. (2011). Characterization of Extracellular Circulating microRNA. Nucleic Acids Res..

[116] Kang E., Jung S.C., Nam S.K., Park Y., Seo S.H., Park K.U., Oh H.-K., Kim D.-W., Kang S.-B., Lee H.S. (2022). Tissue miR-200c-3p and Circulating miR-1290 as Potential Prognostic Biomarkers for Colorectal Cancer. Sci. Rep..

[117] Nassar F.J., Msheik Z.S., Itani M.M., Helou R.E., Hadla R., Kreidieh F., Bejjany R., Mukherji D., Shamseddine A., Nasr R.R. (2021). Circulating miRNA as Biomarkers for Colorectal Cancer Diagnosis and Liver Metastasis. Diagnostics (Basel).

[118] Chen E., Li Q., Wang H., Yang F., Min L., Yang J. (2018). MiR-92a Promotes Tumorigenesis of Colorectal Cancer, a Transcriptomic and Functional Based Study. Biomed. Pharmacother..

[119] Huang Z., Huang D., Ni S., Peng Z., Sheng W., Du X. (2010). Plasma microRNAs Are Promising Novel Biomarkers for Early Detection of Colorectal Cancer. Int. J. Cancer.

[120] Min L., Chen L., Liu S., Yu Y., Guo Q., Li P., Zhu S. (2019). Loss of Circulating Exosomal miR-92b Is a Novel Biomarker of Colorectal Cancer at Early Stage. Int. J. Med. Sci..

[121] Fu F., Jiang W., Zhou L., Chen Z. (2018). Circulating Exosomal miR-17-5p and miR-92a-3p Predict Pathologic Stage and Grade of Colorectal Cancer. Transl. Oncol..

[122] Zou S.-L., Chen Y.-L., Ge Z.-Z., Qu Y.-Y., Cao Y., Kang Z.-X. (2019). Downregulation of Serum Exosomal miR-150-5p Is Associated with Poor Prognosis in Patients with Colorectal Cancer. Cancer Biomark..

[123] Sun L., Liu X., Pan B., Hu X., Zhu Y., Su Y., Guo Z., Zhang G., Xu M., Xu X. (2020). Serum Exosomal miR-122 as a Potential Diagnostic and Prognostic Biomarker of Colorectal Cancer with Liver Metastasis. J. Cancer.

[124] Imaoka H., Toiyama Y., Fujikawa H., Hiro J., Saigusa S., Tanaka K., Inoue Y., Mohri Y., Mori T., Kato T. (2016). Circulating microRNA-1290 as a Novel Diagnostic and Prognostic Biomarker in Human Colorectal Cancer. Ann. Oncol..

[125] Peng X., Wang J., Zhang C., Liu K., Zhao L., Chen X., Huang G., Lai Y. (2020). A Three-miRNA Panel in Serum as a Noninvasive Biomarker for Colorectal Cancer Detection. Int. J. Biol. Markers.

[126] Zhang Y., Li M., Ding Y., Fan Z., Zhang J., Zhang H., Jiang B., Zhu Y. (2017). Serum MicroRNA Profile in Patients with Colon Adenomas or Cancer. BMC Med. Genom..

[127] Maminezhad H., Ghanadian S., Pakravan K., Razmara E., Rouhollah F., Mossahebi-Mohammadi M., Babashah S. (2020). A Panel of Six-Circulating miRNA Signature in Serum and Its Potential Diagnostic Value in Colorectal Cancer. Life Sci..

[128] Sazanov A.A., Kiselyova E.V., Zakharenko A.A., Romanov M.N., Zaraysky M.I. (2017). Plasma and Saliva miR-21 Expression in Colorectal Cancer Patients. J. Appl. Genet..

[129] Rapado-González Ó., Majem B., Álvarez-Castro A., Díaz-Peña R., Abalo A., Suárez-Cabrera L., Gil-Moreno A., Santamaría A., López-López R., Muinelo-Romay L. (2019). A Novel Saliva-Based miRNA Signature for Colorectal Cancer Diagnosis. J. Clin. Med. Res..

[130] Iwasaki H., Shimura T., Kitagawa M., Yamada T., Nishigaki R., Fukusada S., Okuda Y., Katano T., Horike S.-I., Kataoka H. (2022). A Novel Urinary miRNA Biomarker for Early Detection of Colorectal Cancer. Cancers.

[131] Taniue K., Akimitsu N. (2021). The Functions and Unique Features of LncRNAs in Cancer Development and Tumorigenesis. Int. J. Mol. Sci..

[132] Schwarzmueller L., Bril O., Vermeulen L., Léveillé N. (2020). Emerging Role and Therapeutic Potential of lncRNAs in Colorectal Cancer. Cancers.

[133] Galamb O., Barták B.K., Kalmár A., Nagy Z.B., Szigeti K.A., Tulassay Z., Igaz P., Molnár B. (2019). Diagnostic and Prognostic Potential of Tissue and Circulating Long Non-Coding RNAs in Colorectal Tumors. World J. Gastroenterol..

[134] Chen S., Shen X. (2020). Long Noncoding RNAs: Functions and Mechanisms in Colon Cancer. Mol. Cancer.

[135] Wu Y., Yang L., Zhao J., Li C., Nie J., Liu F., Zhuo C., Zheng Y., Li B., Wang Z. (2015). Nuclear-Enriched Abundant Transcript 1 as a Diagnostic and Prognostic Biomarker in Colorectal Cancer. Mol. Cancer.

[136] Dai M., Chen X., Mo S., Li J., Huang Z., Huang S., Xu J., He B., Zou Y., Chen J. (2017). Meta-Signature LncRNAs Serve as Novel Biomarkers for Colorectal Cancer: Integrated Bioinformatics Analysis, Experimental Validation and Diagnostic Evaluation. Sci. Rep..

[137] Shaker O.G., Senousy M.A., Elbaz E.M. (2017). Association of rs6983267 at 8q24, HULC rs7763881 Polymorphisms and Serum lncRNAs CCAT2 and HULC with Colorectal Cancer in Egyptian Patients. Sci. Rep..

[138] Wang L., Duan W., Yan S., Xie Y., Wang C. (2019). Circulating Long Non-Coding RNA Colon Cancer-Associated Transcript 2 Protected by Exosome as a Potential Biomarker for Colorectal Cancer. Biomed. Pharmacother..

[139] Liu T., Zhang X., Gao S., Jing F., Yang Y., Du L., Zheng G., Li P., Li C., Wang C. (2016). Exosomal Long Noncoding RNA CRNDE-H as a Novel Serum-Based Biomarker for Diagnosis and Prognosis of Colorectal Cancer. Oncotarget.

[140] Yang Y.-N., Zhang R., Du J.-W., Yuan H.-H., Li Y.-J., Wei X.-L., Du X.-X., Jiang S.-L., Han Y. (2018). Predictive Role of UCA1-Containing Exosomes in Cetuximab-Resistant Colorectal Cancer. Cancer Cell Int..

[141] Yu M., Song X.-G., Zhao Y.-J., Dong X.-H., Niu L.-M., Zhang Z.-J., Shang X.-L., Tang Y.-Y., Song X.-R., Xie L. (2021). Circulating Serum Exosomal Long Non-Coding RNAs FOXD2-AS1, NRIR, and XLOC_009459 as Diagnostic Biomarkers for Colorectal Cancer. Front. Oncol..

[142] Abedini P., Fattahi A., Agah S., Talebi A., Beygi A.H., Amini S.M., Mirzaei A., Akbari A. (2019). Expression Analysis of Circulating Plasma Long Noncoding RNAs in Colorectal Cancer: The Relevance of lncRNAs ATB and CCAT1 as Potential Clinical Hallmarks. J. Cell. Physiol..

[143] Zhao W., Song M., Zhang J., Kuerban M., Wang H. (2015). Combined Identification of Long Non-Coding RNA CCAT1 and HOTAIR in Serum as an Effective Screening for Colorectal Carcinoma. Int. J. Clin. Exp. Pathol..

[144] Liu Y., Chen X., Chen X., Liu J., Gu H., Fan R., Ge H. (2020). Long Non-Coding RNA HOTAIR Knockdown Enhances Radiosensitivity through Regulating microRNA-93/ATG12 Axis in Colorectal Cancer. Cell Death Dis..

[145] Hu D., Zhan Y., Zhu K., Bai M., Han J., Si Y., Zhang H., Kong D. (2018). Plasma Exosomal Long Non-Coding RNAs Serve as Biomarkers for Early Detection of Colorectal Cancer. Cell. Physiol. Biochem..

[146] Zhu M., Luo Y., Xu A., Xu X., Zhong M., Ran Z. (2020). Long Noncoding RNA TCONS_00026334 Is Involved in Suppressing the Progression of Colorectal Cancer by Regulating miR-548n/TP53INP1 Signaling Pathway. Cancer Med..

[147] Xiao W., Li J., Hu J., Wang L., Huang J.-R., Sethi G., Ma Z. (2021). Circular RNAs in Cell Cycle Regulation: Mechanisms to Clinical Significance. Cell Prolif..

[148] Ameli-Mojarad M., Ameli-Mojarad M., Hadizadeh M., Young C., Babini H., Nazemalhosseini-Mojarad E., Bonab M.A. (2021). The Effective Function of Circular RNA in Colorectal Cancer. Cancer Cell Int..

[149] Memczak S., Jens M., Elefsinioti A., Torti F., Krueger J., Rybak A., Maier L., Mackowiak S.D., Gregersen L.H., Munschauer M. (2013). Circular RNAs Are a Large Class of Animal RNAs with Regulatory Potency. Nature.

[150] Ren S., Lin P., Wang J., Yu H., Lv T., Sun L., Du G. (2020). Circular RNAs: Promising Molecular Biomarkers of Human Aging-Related Diseases via Functioning as an miRNA Sponge. Mol. Ther. Methods Clin. Dev..

[151] Liu K., Mou Y., Shi X., Liu T., Chen Z., Zuo X. (2021). Circular RNA 100146 Promotes Colorectal Cancer Progression by the MicroRNA 149/HMGA2 Axis. Mol. Cell. Biol..

[152] Li S., Yan G., Liu W., Li C., Wang X. (2020). Circ0106714 Inhibits Tumorigenesis of Colorectal Cancer by Sponging miR-942-5p and Releasing DLG2 via Hippo-YAP Signaling. Mol. Carcinog..

[153] Xu X.-W., Zheng B.-A., Hu Z.-M., Qian Z.-Y., Huang C.-J., Liu X.-Q., Wu W.-D. (2017). Circular RNA hsa_circ_000984 Promotes Colon Cancer Growth and Metastasis by Sponging miR-106b. Oncotarget.

[154] Yu C., Li D., Yan Q., Wang Y., Yang X., Zhang S., Zhang Y., Zhang Z. (2021). Circ_0005927 Inhibits the Progression of Colorectal Cancer by Regulating miR-942-5p/BATF2 Axis. Cancer Manag. Res..

[155] Shang A., Gu C., Wang W., Wang X., Sun J., Zeng B., Chen C., Chang W., Ping Y., Ji P. (2020). Exosomal circPACRGL Promotes Progression of Colorectal Cancer via the miR-142-3p/miR-506-3p- TGF-β1 Axis. Mol. Cancer.

[156] Geng Y., Zheng X., Hu W., Wang Q., Xu Y., He W., Wu C., Zhu D., Wu C., Jiang J. (2019). Hsa_circ_0009361 Acts as the Sponge of miR-582 to Suppress Colorectal Cancer Progression by Regulating APC2 Expression. Clin. Sci..

[157] Hsiao K.-Y., Lin Y.-C., Gupta S.K., Chang N., Yen L., Sun H.S., Tsai S.-J. (2017). Noncoding Effects of Circular RNA CCDC66 Promote Colon Cancer Growth and Metastasis. Cancer Res..

[158] Lu H., Yao B., Wen X., Jia B. (2019). FBXW7 Circular RNA Regulates Proliferation, Migration and Invasion of Colorectal Carcinoma through NEK2, mTOR, and PTEN Signaling Pathways in Vitro and in Vivo. BMC Cancer.

[159] Ren C., Zhang Z., Wang S., Zhu W., Zheng P., Wang W. (2020). Circular RNA hsa_circ_0001178 Facilitates the Invasion and Metastasis of Colorectal Cancer through Upregulating ZEB1 via Sponging Multiple miRNAs. Biol. Chem..

[160] Ren T.-J., Liu C., Hou J.-F., Shan F.-X. (2020). CircDDX17 Reduces 5-Fluorouracil Resistance and Hinders Tumorigenesis in Colorectal Cancer by Regulating miR-31-5p/KANK1 Axis. Eur. Rev. Med. Pharmacol. Sci..

[161] Wang X., Zhang H., Yang H., Bai M., Ning T., Deng T., Liu R., Fan Q., Zhu K., Li J. (2020). Exosome-Delivered circRNA Promotes Glycolysis to Induce Chemoresistance through the miR-122-PKM2 Axis in Colorectal Cancer. Mol. Oncol..

[162] Jian X., He H., Zhu J., Zhang Q., Zheng Z., Liang X., Chen L., Yang M., Peng K., Zhang Z. (2020). Hsa_circ_001680 Affects the Proliferation and Migration of CRC and Mediates Its Chemoresistance by Regulating BMI1 through miR-340. Mol. Cancer.

[163] Xu Y., Qiu A., Peng F., Tan X., Wang J., Gong X. (2021). Exosomal Transfer of Circular RNA FBXW7 Ameliorates the Chemoresistance to Oxaliplatin in Colorectal Cancer by Sponging miR-18b-5p. Neoplasma.

[164] Xi L., Liu Q., Zhang W., Luo L., Song J., Liu R., Wei S., Wang Y. (2021). Circular RNA circCSPP1 Knockdown Attenuates Doxorubicin Resistance and Suppresses Tumor Progression of Colorectal Cancer via miR-944/FZD7 Axis. Cancer Cell Int..

[165] Zhao K., Cheng X., Ye Z., Li Y., Peng W., Wu Y., Xing C. (2021). Exosome-Mediated Transfer of circ_0000338 Enhances 5-Fluorouracil Resistance in Colorectal Cancer through Regulating MicroRNA 217 (miR-217) and miR-485-3p. Mol. Cell. Biol..

[166] Hon K.W., Ab-Mutalib N.S., Abdullah N.M.A., Jamal R., Abu N. (2019). Extracellular Vesicle-Derived Circular RNAs Confers Chemoresistance in Colorectal Cancer. Sci. Rep..

[167] Yu C.-Y., Kuo H.-C. (2019). The Emerging Roles and Functions of Circular RNAs and Their Generation. J. Biomed. Sci..

[168] Liang Y., Shi J., He Q., Sun G., Gao L., Ye J., Tang X., Qu H. (2020). Hsa_circ_0026416 Promotes Proliferation and Migration in Colorectal Cancer via miR-346/NFIB Axis. Cancer Cell Int..

[169] Mohammadi D., Zafari Y., Estaki Z., Mehrabi M., Moghbelinejad S. (2022). Evaluation of Plasma circ_0006282 as a Novel Diagnostic Biomarker in Colorectal Cancer. J. Clin. Lab. Anal..

[170] Li J., Song Y., Wang J., Huang J. (2020). Plasma Circular RNA Panel Acts as a Novel Diagnostic Biomarker for Colorectal Cancer Detection. Am. J. Transl. Res..

[171] Ye D.-X., Wang S.-S., Huang Y., Chi P. (2019). A 3-Circular RNA Signature as a Noninvasive Biomarker for Diagnosis of Colorectal Cancer. Cancer Cell Int..

[172] Pan B., Qin J., Liu X., He B., Wang X., Pan Y., Sun H., Xu T., Xu M., Chen X. (2019). Identification of Serum Exosomal Hsa-Circ-0004771 as a Novel Diagnostic Biomarker of Colorectal Cancer. Front. Genet..

[173] Zhang J., Cai A., Zhao Y. (2020). Three CircRNAs Function as Potential Biomarkers for Colorectal Cancer. Clin. Lab..

[174] Sobhani I., Tap J., Roudot-Thoraval F., Roperch J.P., Letulle S., Langella P., Corthier G., Tran Van Nhieu J., Furet J.P. (2011). Microbial Dysbiosis in Colorectal Cancer (CRC) Patients. PLoS ONE.

[175] Rezasoltani S., Asadzadeh-Aghdaei H., Nazemalhosseini-Mojarad E., Dabiri H., Ghanbari R., Zali M.R. (2017). Gut Microbiota, Epigenetic Modification and Colorectal Cancer. Iran. J. Microbiol..

[176] Marchesi J.R., Dutilh B.E., Hall N., Peters W.H.M., Roelofs R., Boleij A., Tjalsma H. (2011). Towards the Human Colorectal Cancer Microbiome. PLoS ONE.

[177] Xiao Q., Lu W., Kong X., Shao Y.W., Hu Y., Wang A., Bao H., Cao R., Liu K., Wang X. (2021). Alterations of Circulating Bacterial DNA in Colorectal Cancer and Adenoma: A Proof-of-Concept Study. Cancer Lett..

